# Quantification of water and lipid composition of perivascular adipose tissue using coronary CT angiography: a simulation study

**DOI:** 10.1007/s10554-025-03390-1

**Published:** 2025-04-10

**Authors:** Shu Nie, Sabee Molloi

**Affiliations:** 1 Department of Radiological Sciences, University of California, Irvine, Irvine, CA 92697 USA; 2 Department of Radiological Sciences, Medical Sciences I, B-140, University of California, Irvine, CA 92697 USA

**Keywords:** CT number, Perivascular adipose tissue (PVAT), Material decomposition

## Abstract

Early detection of vascular inflammation via perivascular adipose tissue (PVAT) compositional changes (e.g., increased water content) could improve cardiovascular risk stratification. However, CT-based measurements face variability due to tube voltage and patient size. This study aims to quantify perivascular adipose tissue (PVAT) composition (water, lipid, protein) using coronary CT angiography and assess impacts of tube voltage, patient size, and positional variability on measurements. A 320-slice CT simulation generated anthropomorphic thorax phantoms (small, medium, large) with fat rings mimicking different patient sizes. Ten randomized water-lipid-protein inserts were placed within the thorax phantom. Three-material decomposition was applied using medium phantoms with different tube voltages and different patient sizes at 120 kV. PVAT CT number (HU) increased with higher tube voltages and larger patient sizes. The root-mean-squared errors (RMSE) for water volumetric fraction measurements were 0.26%, 0.64%, 0.01%, and 0.15% for 80, 100, 120, and 135 kV, respectively, and 0.19%, 0.35%, and 0.61% for small, medium, and large size phantoms at 120 kV, respectively. The root-mean-squared deviations (RMSD) were 3.52%, 2.94%, 4.96%, and 6.00% for 80, 100, 120, and 135 kV, respectively, and 3.82%, 3.74%, and 6.05% for small, medium, and large size phantoms at 120 kV, respectively. Clinically relevant water fractions spanned 17–37%, with inflammation expected to alter values by approximately 5%. The findings of this study indicate that, after accounting for the effects of tube voltage and patient size, perivascular adipose tissue CT number can be quantitatively represented in terms of its water composition. This decomposition method has the potential to enable quantification of water composition and facilitate early detection of coronary artery inflammation.

## Introduction

Perivascular adipose tissue (PVAT) dysfunction, resulting from coronary inflammation, plays a crucial role in various vascular diseases, including vascular aging, atherogenesis, and hypertension [1]. In healthy individuals, PVAT has anti-atherogenic properties that protect against the formation of detrimental arterial plaques [2, 3]. However, under pathological conditions, dysfunctional PVAT loses its ability to store lipids and instead releases pro-inflammatory cytokines and chemokines, contributing to cardiovascular inflammation [4]. Early detection of such inflammation is expected to improve cardiovascular risk stratification and enable effective prevention and treatment strategies for different disease states [5].

Recent studies have demonstrated that coronary computed tomography angiography (CCTA) can detect perivascular lipid accumulation through the measurement of the fat attenuation index (FAI) in Hounsfield units (HU) [6]. Some studies have also reported higher fat attenuation index values in patients with elevated cardiovascular risk [7]. However, several limitations exist. Firstly, the initial studies were conducted using a fixed tube voltage of 120 kV, while it is known that the HU value changes with different tube voltages used during coronary CT angiography acquisition [8]. Although recent reports have introduced conversion factors to correct HU measurements for tube voltages other than 120 kV, the correction remains limited in accurately determining changes in lipid content [9]. Furthermore, PVAT HU is also changed by its position and the size of the patient. Previous studies have reported a ≤ 5 HU difference in PVAT CT number between healthy and patients with coronary artery disease at 120 kV, suggesting biologically meaningful compositional changes [6]. However, the exact water-fraction change corresponding to this HU difference is unknown. Unlike HU values—which are confounded by technical factors like tube voltage and patient size—water-fraction provides a stable, intrinsic tissue property directly reflecting PVAT composition. Quantifying water-fraction could therefore serve as a more reliable biomarker for vascular inflammation.

To address these gaps, we developed a comprehensive simulation framework to systematically evaluate and correct for these confounding factors in CT number measurements. This study introduces a novel approach using a three-material decomposition framework, which quantitatively assesses PVAT composition to overcome these limitations.

## Methods

### CT simulation

A CT simulation package was developed to replicate the scanning parameters of a 320-slice CT scanner (Canon Aquilion One, Canon America Medical Systems, Tustin, CA), as reported in a previous study [10]. Simulated single-slice CT images were reconstructed with Filtered Back Projection (FBP) using a Ram-Lak filter and were augmented with Poisson noise to mimic the noise characteristics of real-world images [11]. Source-to-Object Distance (SOD) and Source-to-Detector Distance (SDD) were specified to 600 cm and 1072 cm, respectively. Calibration and validation methods were performed to adjust the exposure value in mR utilized in the CT simulation.

The detailed acquisition and reconstruction parameters are shown in Table 1.

**Table 1:**
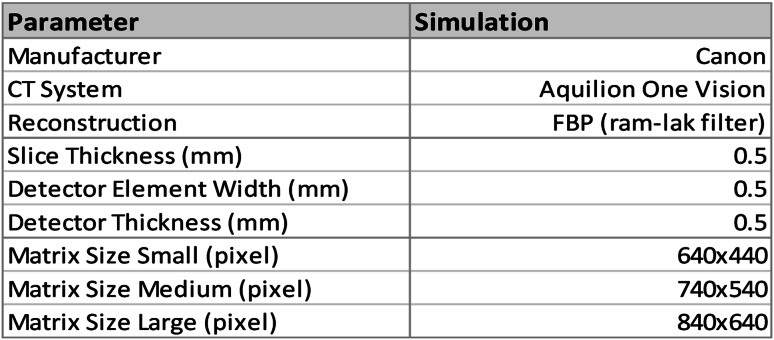
Acquisition and reconstruction parameters of the simulation

**Fig. 1:**
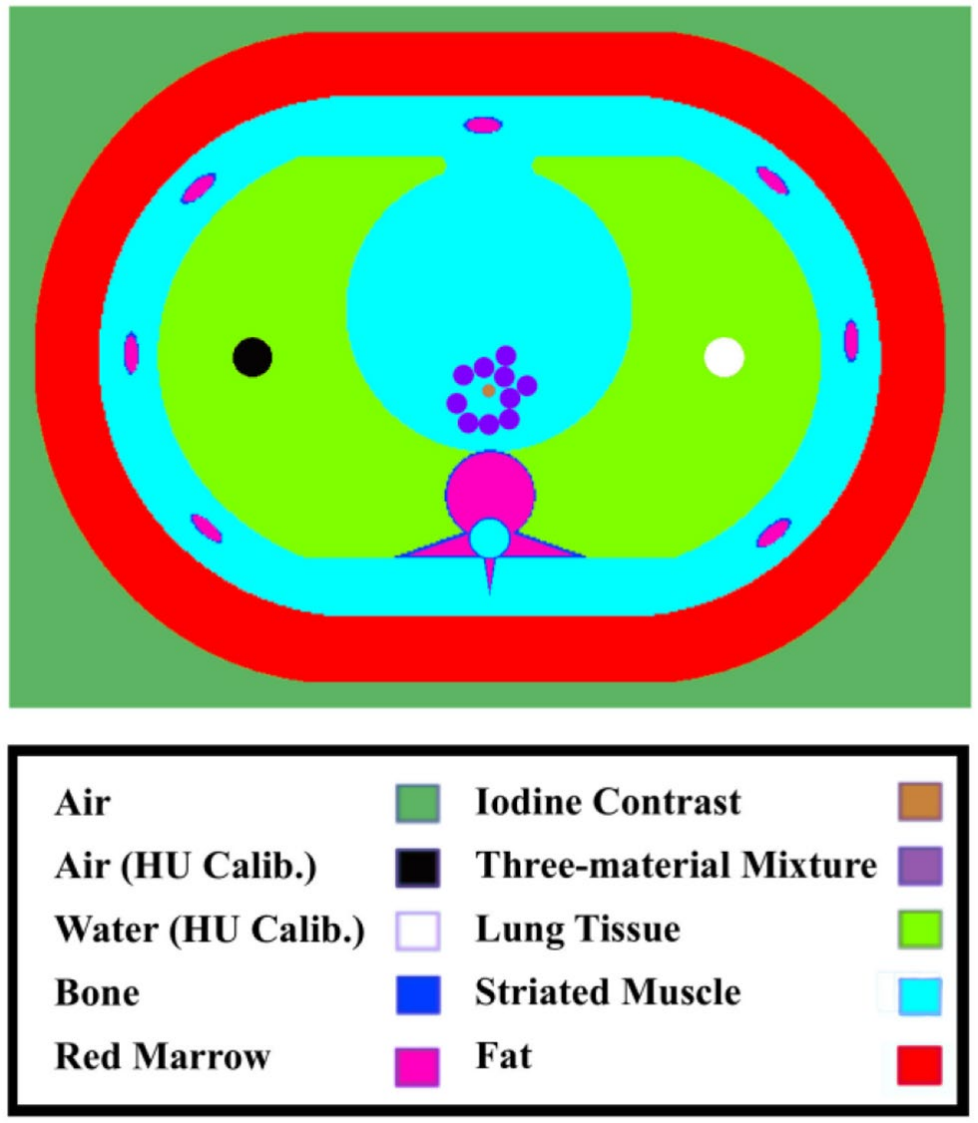
Shows a sketch of the simulated phantom with the colors highlighting the different materials in the simulated phantom. Note that the position of three-material inserts is randomly generated without overlap

**Fig. 2:**
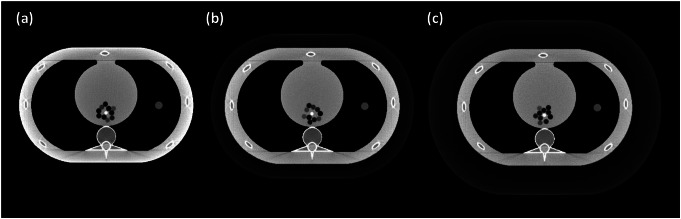
Shows simulated phantom images of small (**a**) medium (**b**) and large (**c**) phantom sizes

In this simulation study, a 3200 × 2200 pixels digital phantom was constructed based on a standard, commercially available anthropomorphic thorax phantom with a size of 30 × 20 cm^2^ (QRM-Thorax, QRM, Mӧhrendorf, Germany). To evaluate material decomposition accuracy under clinically representative conditions, two complementary phantom configurations were implemented. The first one is iodine-contrast phantom for material decomposition. Ten 8 mm diameter circular inserts composed of water, lipid, and protein were randomly placed within the thorax (Figs. 1 and 2). To minimize partial volume effect, a 5.6 mm diameter region of interest (ROI), located 3 pixels away from the vessel’s edge was used for measurements. Mean CT number (HU) within each ROI was calculated to quantify material-specific CT values.

**Fig. 3:**
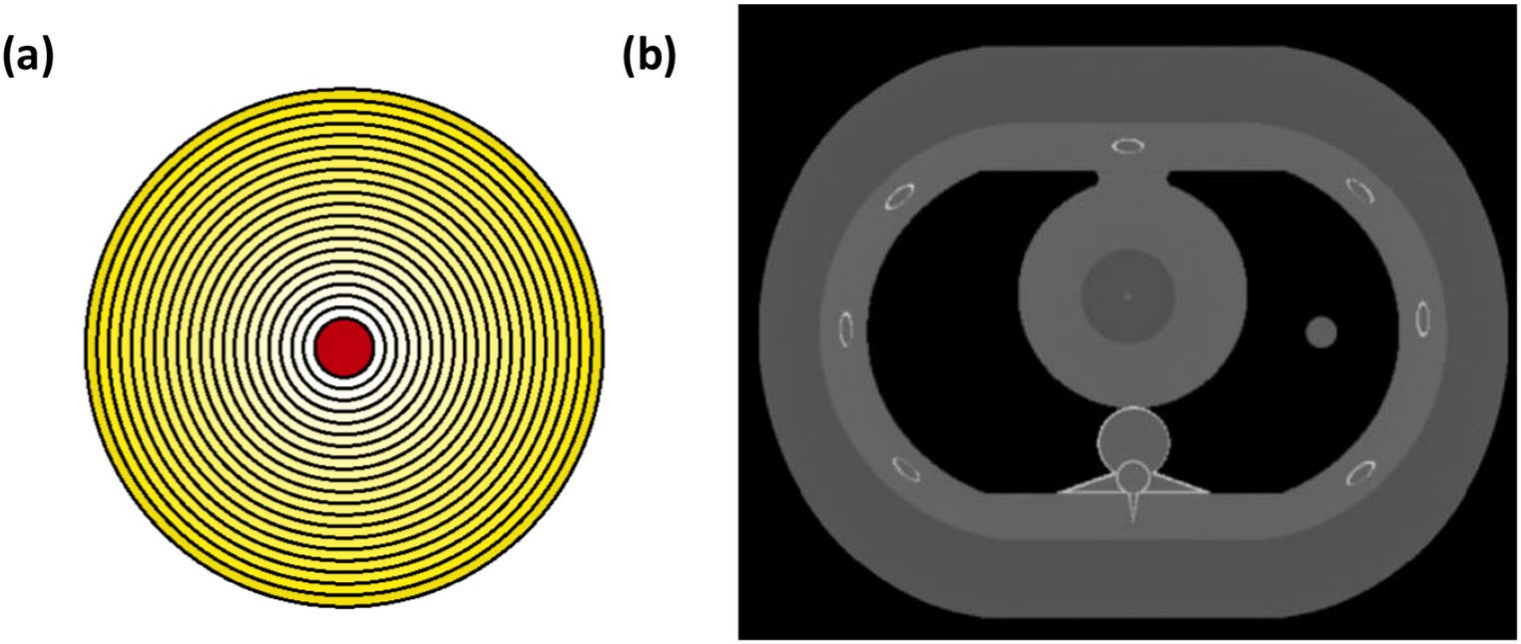
(**a**) Design of the concentric cylinder phantom: central vessel (3 mm diameter) surrounded by 20 PVAT rings (1 mm thick). Colors indicate the concentration of lipids (yellow = lipid, white = water). (**b**) Simulated concentric cylinder phantom within a chest phantom

Next, in Fig. 3, a concentric cylindrical phantom was created to simulate the CT number contrast between a non-contrast coronary artery (3 mm diameter) and surrounding perivascular adipose tissue (PVAT), modeled as 20 concentric rings (1 mm thickness per ring, total radial thickness = 20 mm) with spatially varying lipid-water ratios. To compare physiological and pathological states, two configurations were simulated: healthy PVAT (higher lipid content, lower water fraction) and coronary artery diseased PVAT (elevated water fraction mimicking inflammation). The phantom was placed within a thorax phantom, and radial HU profiles were calculated from the arterial wall outward (2–19 mm range) to minimize partial volume effects.

In the experiment, we performed volumetric fraction calculations of water, lipid, and protein using MATLAB. Volumetric fraction refers to the fraction of the total volume of a composite material that is occupied by a particular component. This parameter is commonly used to characterize the microstructure and properties of composite materials, which are made up of multiple components that are mixed on a microscopic scale.

### Experimental design

In this study, a comprehensive calibration experiment was undertaken to investigate the influence of tube voltage and patient size on the linear relationship between water volumetric fractions and the average HU derived from the region of interest in the CT images. We systematically examined the effects of tube voltage using a medium-sized phantom (35 × 25 cm²), adjusting the tube voltage across four levels: 80, 100, 120, and 135 kilovolts (kV). For the patient size analysis, small, medium, and large phantoms were scanned with a tube voltage of 120 kV to isolate the effect of patient size on the water volume fraction measurements. In each of these settings, five images were produced, yielding a total of 50 unique samples for the calibration set. For calibration purposes, high dose images (500 mR) were used to minimize the image noise (2.47 HU ± 0.33).

To simulate different patient sizes, we utilized extension fat rings, generating medium-sized (35 × 25 cm²) and large-sized (40 × 30 cm²) phantoms. These fat rings were designed to mimic the subcutaneous fat layer. To approximate the composition of adipose tissue, the layer was simulated using a mixture of 19.08% water, 78.75% lipid, and 2.17% protein by volume, closely corresponding to the reported mass composition of adipose tissue: 20.2% water, 76.7% lipid, and 3.1% protein [12].

This study conducted validation experiments to assess the accuracy of the established calibration methods. The noise level, a crucial factor in image analysis, was fine-tuned to better replicate the noise for coronary CT angiography. This adjustment aimed to ensure that the validation process for tube voltage analysis reflects as closely as possible the realistic image noise level, specifically 18.1 HU ± 1.69, typically found in coronary CT angiography images [13].

For patient size analysis, water volumetric fractions were quantified, with a focus on understanding the potential calibration error across different patient sizes. Patient sizes were categorized based on the sum of lateral width (LAT) and anteroposterior thickness (AP), with ranges of 50–56 cm for small, 57–63 cm for medium, and 64–70 cm for large. The calibration was tested on phantom sizes that fall in between the standard small, medium, and large sizes. The calibration lines derived from the study were then applied to estimate the volumetric fractions of the water component in 50 inserts, and the estimated values were compared with the actual fractions. The study tested a range of water (0–97.83%), lipid (0–97.83%), and protein (fixed at 2.17%) based on reported compositions of adipose tissue [12]. It is assumed that the volumetric fraction of protein remains constant at 2.17% in the simulation, with only water and lipid considered as variables. Radiation dose was adjusted based on the sum of LAT and AP [14]. A polynomial regression model was used to fit the exposure values used for standard small, medium, and large phantom sizes (Eq. 1). Detailed exposure information can be found in the Table 2 below [15].

**Table 2:**
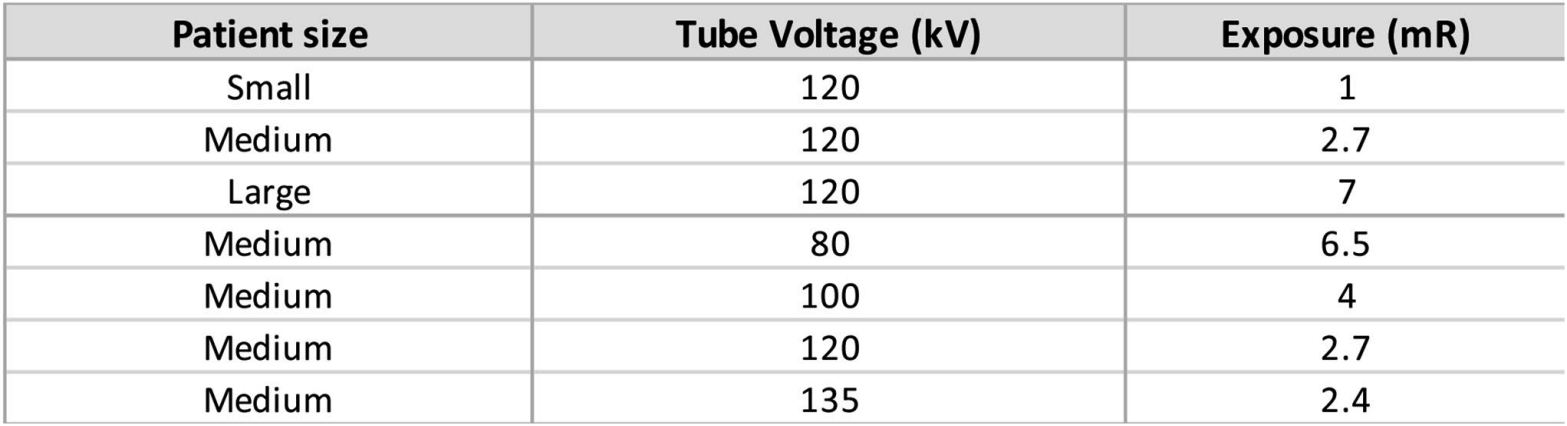
Radiation exposure levels measured in milliroentgens (mR) across patient sizes and tube voltages, calculated using eq. 1 using lateral width (LAT) and anteroposterior thickness (AP) in cm

1 $$\text{Exposure}=0.0014(LAT+AP)^2-0.38(LAT+AP)+27.68$$

### Linear regression and statistical analysis

The linear relationship between water volumetric fractions and CT number was analyzed using statistical methods in Microsoft Excel, and the root-mean-square error (RMSE) and root-mean-square deviation (RMSD) were calculated to test for accuracy and precision, respectively. The RMSE was used to measure the error between the measured values and known values (Eq. 2), while the RMSD measured the variance between the measured values and the fitted regression lines on the plots (Eq. 3). The number of data points, represented by *N*, was used in both equations to normalize the results.

2 $$\:RMSE=\sqrt{\frac{\sum\:_{i=1}^{N}{({Measured}_{i}-{Known}_{i})}^{2}}{N}}$$

3 $$\:RMSD=\sqrt{\frac{\sum\:_{i=1}^{N}{({Measured}_{i}-{Fitted}_{i})}^{2}}{N}}$$

## Results

### Calibration results

**Fig. 4:**
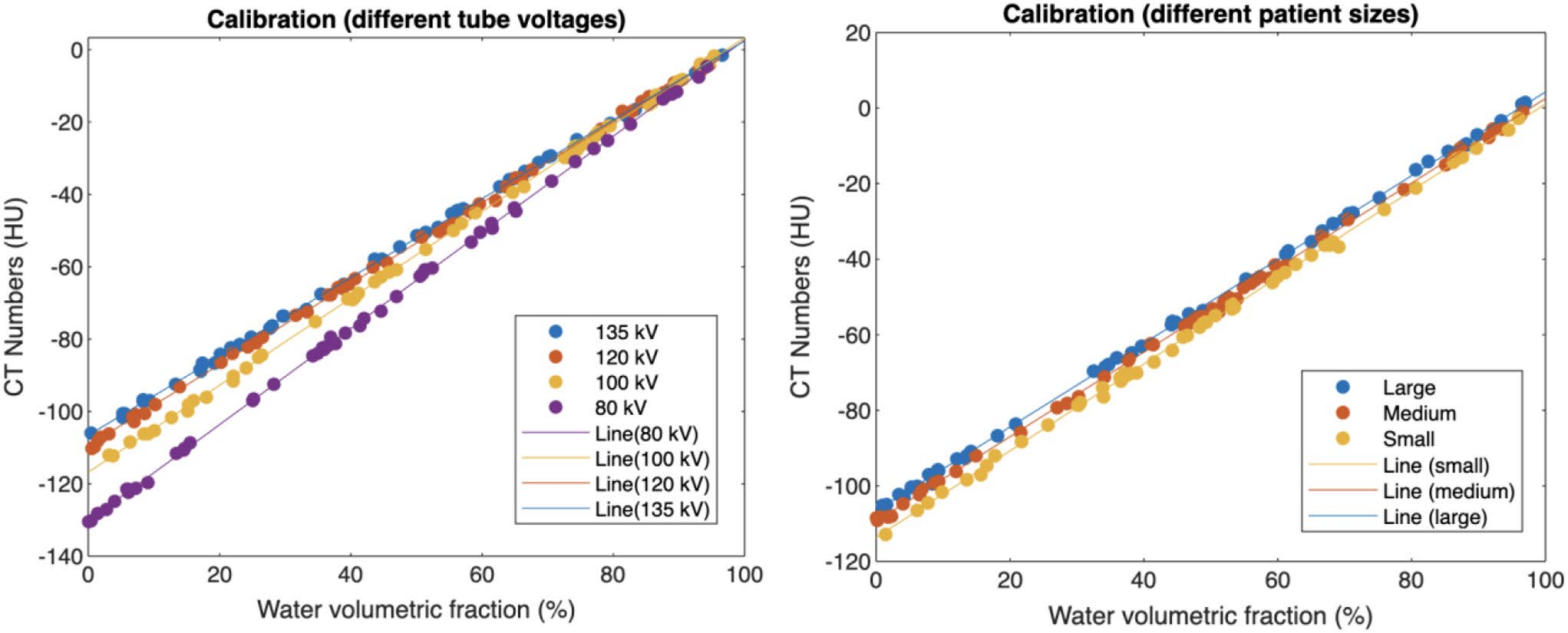
Impact of tube voltage (**a**) and patient size (**b**) on calibration slope. (**a**) Calibration slope decreases with higher tube voltages (reduced energy-dependent CT number), with regression lines converging at 0 HU (100% water). (**b**) Slope stability across patient sizes contrasts with baseline CT number shifts (HU intercept: large > medium > small)

The calibration phase aimed to establish accurate baseline material-specific CT number properties across different tube voltages and patient sizes. The results of the calibration process, as visualized in the linear graphs of water volumetric fraction (%) versus CT number (HU) for both tube voltage and patient size, demonstrated clear patterns. The linear relationships between these variables can be observed in Figures 4, with the respective slopes and intercepts providing important insights into their interplay (Table 3).

Notably, the slope ratio to the standard 120 kV/medium conditions and the conversion factors further elucidated the impact of these variables on the water volumetric fraction. Moreover, we have derived a conversion factor to adjust the CT number due to different tube voltages and patient sizes. The conversion factor is determined by the ratio of the slopes of the linear relationship between water volumetric fraction and CT number at the condition of interest to the standard condition (120 kV and medium size). Mathematically, this can be represented as:

4 $${Conversion}\,{Factor}_{\left(Energy,\,Size\right)}=\frac{{Slope}_{(Energy,\:Size)}}{{Slope}_{(120\:kV,\:Medium)}}$$

**Table 3:**
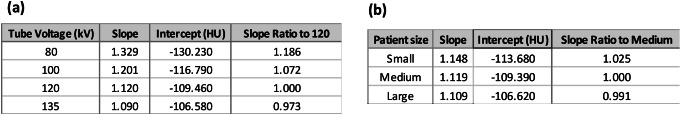
Linear regression parameters (slope, intercept) for CT number (HU) versus water volumetric fraction (%), evaluated at different tube voltages (a) and patient sizes (b)

**Fig. 5:**
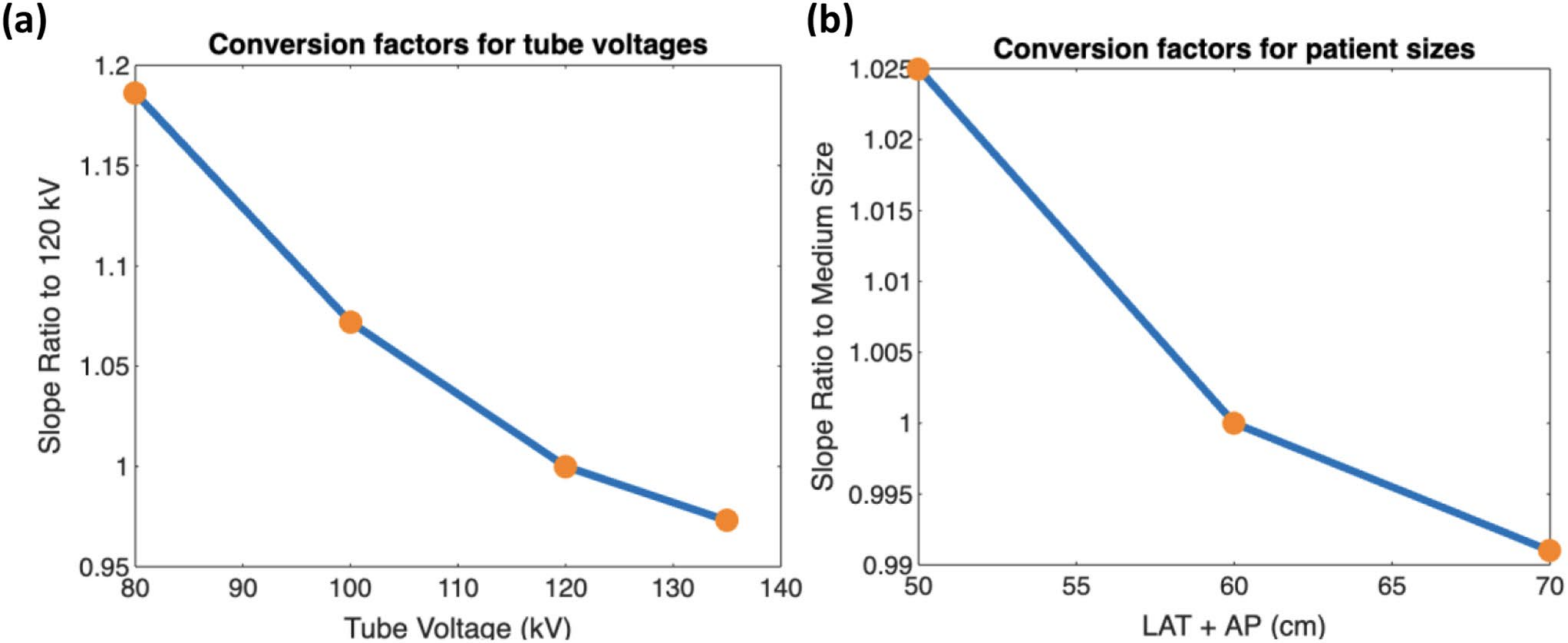
Conversion factors for different tube voltages (**a**) and patient sizes (**b**) based on calibration slope ratios. The patient size was calculated by the sum of lateral width (LAT) and anteroposterior thickness (AP). The effect of tube voltage is more significant than patient size

We have also visualized the conversion factors for both tube voltage and patient size scenarios in two separate line graphs. In these graphs, the x-axis represents the tube voltage (80, 100, 120, and 135 kV) for the tube voltage analysis, and patient size (small, medium, and large) for the patient size analysis. The y-axis corresponds to the conversion factor value in Figure 5.

### Validation results

The validation phase tested the framework’s robustness against ground-truth PVAT compositions under realistic noise conditions.

#### Tube voltage analysis

**Fig. 6:**
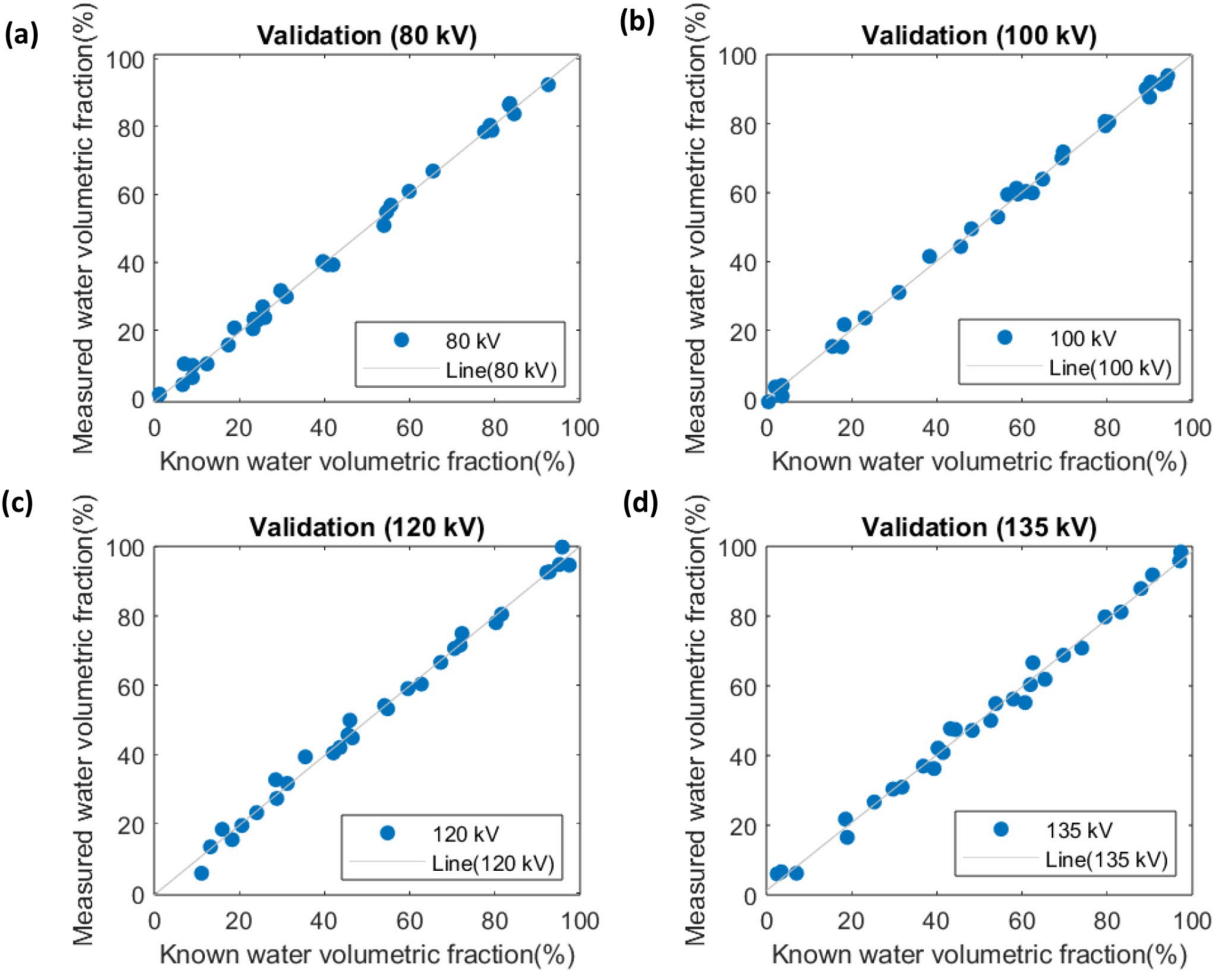
Validations for water volumetric fractions at different tube voltages at 80 kV (**a**), 100 kV (**b**), 120 kV (**c**), and 135 kV (**d**). Measured vs. known values demonstrate strong agreement, confirming calibration accuracy

In the tube voltage validation, we used the calibration lines to estimate the water volumetric fractions in 30 newly generated inserts. These estimates were then compared with the known water volumetric fractions, which were derived from Hounsfield Unit (HU) values after adjusting the radiation dose to simulate realistic image noise levels. The comparison between measured and known values is presented in Fig. 6. A summary of the linear regression parameters is shown in Table 3.

**Table 4:**
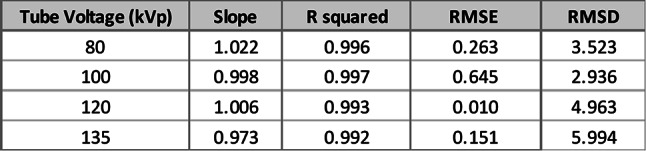
Validation of linear regression models across different tube voltages (medium-sized phantom). Performance metrics include coefficient of determination (R²), root mean square error (RMSE), and root mean square deviation (RMSD) for water volumetric fraction estimation

#### Patient size analysis

**Fig. 7:**
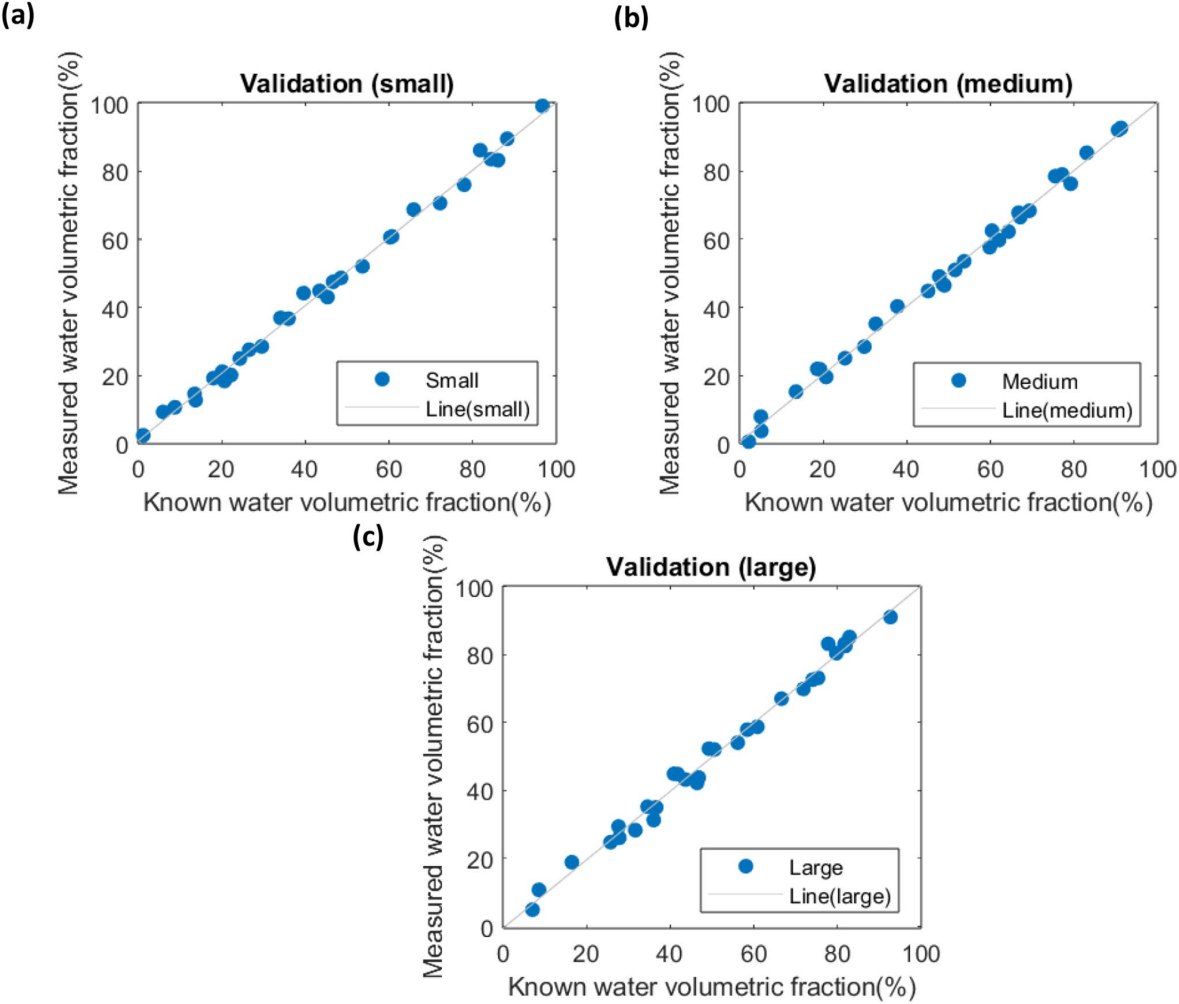
Validations for water volumetric factions for different patient sizes including small (**a**), medium (**b**), and large (**c**)

For patient size validation, 9 random-sized phantoms were built to test the calibration (Table 5). We again applied the calibration lines to estimate water volumetric fractions. The estimated values were compared to the actual fractions. The results for different patient sizes are displayed in Fig. 7. A summary of the linear regression parameters is shown in Table 4.

**Table 5:**
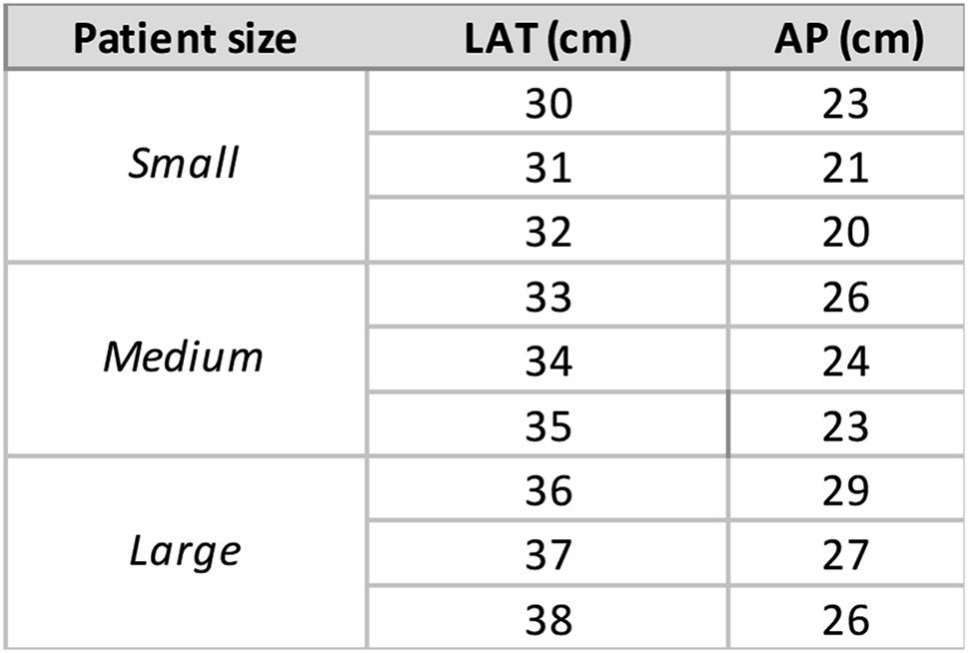
Summary of the lateral width (LAT) and anteroposterior thickness (AP) for each phantom tested for different patient sizes

**Table 6:**
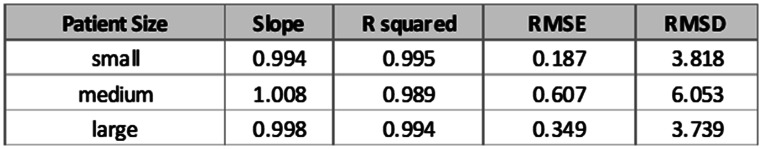
Validation of linear regression models across different patient sizes (at 120 kV). Metrics include R², RMSE, and RMSD

**Table 7:**
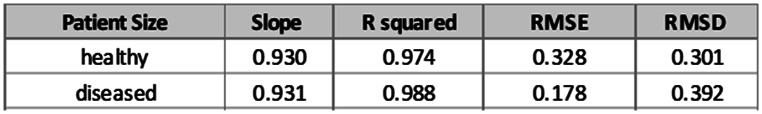
Summary of the linear regression parameters for validations in concentric cylinder Phantom. All the validation measurements were made at 120 kv

#### Water volumetric fraction estimates in healthy and diseased States

**Fig. 8:**
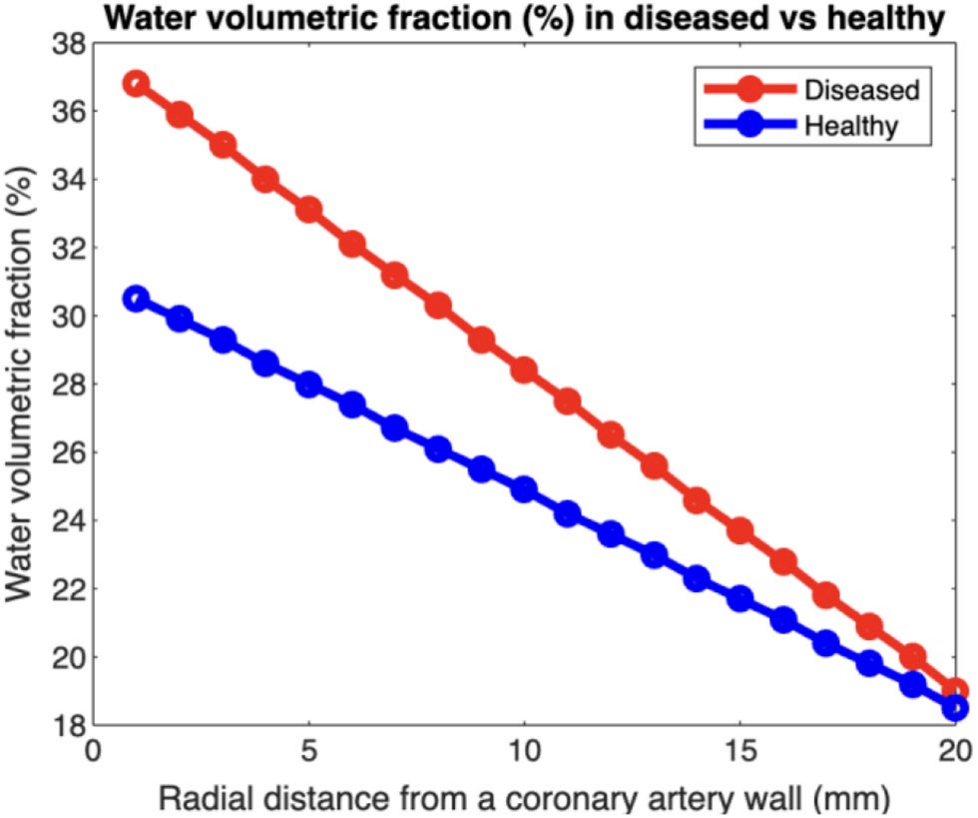
Simulated water volumetric fractions with respect to radial distance from a coronary artery outer wall for diseased and healthy individuals

Water volumetric fraction was estimated using HU values (medium size at 120 kV) derived from a previous study by A. Antonopoulos et al. [6]. This study reported the range of fat attenuation index in HU for healthy individuals and those with coronary artery disease. In this case, the water volumetric fraction ranged from 40 to 17%, corresponding to HU values between − 65 and − 90. Using our calibration method, this HU difference translates to a water volumetric fraction range of 20–30% for healthy individuals and 20–35% for patients with coronary artery disease (Fig. 8), corresponding to an approximate 5% increase in water fraction due to vascular inflammation. This threshold aligns with biologically plausible compositional shifts in PVAT. The measured RMSE (0.19–0.61%) and RMSD (3.74–6.05%) in the previous validation experiments indicate that the method achieves sufficient accuracy to resolve the 5% clinical threshold (RMSE < 2.5%) but requires protocol optimization to reduce variability (RMSD) for robust clinical classification.

**Fig. 9:**
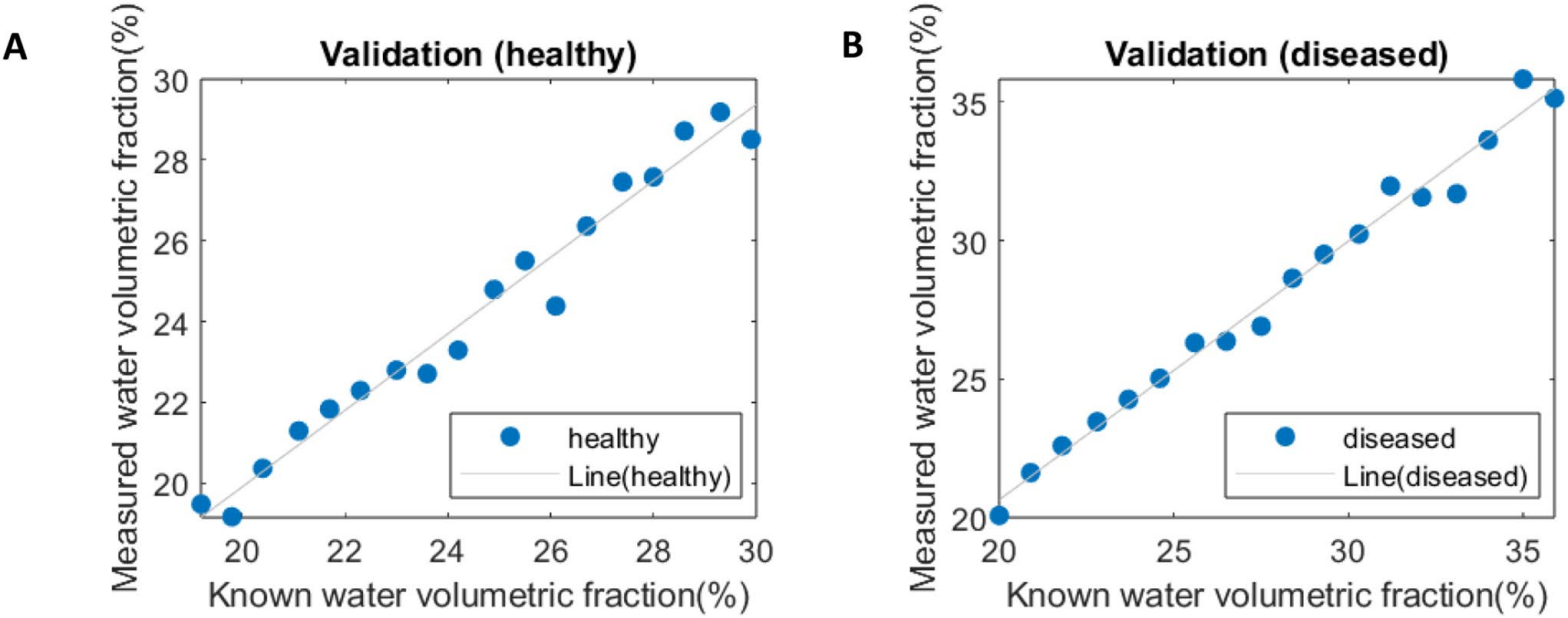
Concentric cylinder phantom validations for water volumetric factions for healthy and diseased

In the concentric cylinder phantom, HU values were first measured radially around the blood vessel. Radial distance in the phantom directly mimics the anatomical gradation of PVAT composition (Fig. 3a), where layers closer to the vessel wall contain more water due to inflammatory infiltration, while outer layers incorporate more lipid-rich adipose tissue [4]. These HU values were then used to calculate the corresponding water volumetric fractions. Subsequently, these calculated fractions were compared with the predetermined water volumetric fractions, as illustrated in Fig. 8. The known versus measured values in healthy versus diseased conditions was compared in Fig. 9. Linear regression parameters, detailed in Table 5, underscore the accuracy and reliability of our measurements, demonstrating strong correlations (R² values of 0.974 and 0.988 for healthy and diseased conditions, respectively) and low error margins as indicated by RMSE and RMSD values.

## Discussion

The present study provides a methodology for detecting coronary inflammation with quantification of water content in adipose tissue, as well as assessing the impact of tube voltages and patient sizes on CT number. The results indicate that CT number did not significantly change for different patient sizes. As expected, the differences in CT numbers for different tube voltages were larger than those for the patient sizes. The results indicate that CT numbers were influenced by both tube voltages and patient sizes, with an extreme variance of 21.9% observed between 80 kV and 135 kV, and a more minor variance of 3.6% between small and large patient sizes.

A previous report has demonstrated that pericoronary adipose tissue mean HU increases linearly with higher tube voltages, with pericoronary adipose tissue mean CT number values of -95.6 HU at 70 kV, -90.2 HU at 80 kV, -87.3 HU at 90 kV, -82.7 HU at 100 kV, and − 79.3 HU at 120 kV [16]. This trend suggests that without standardizing tube voltage, fat attenuation index measurements may not be comparable across studies or clinical assessments. Notably, a 5% HU value discrepancy may arise if tube voltages are changed between 100 and 120 kV [5]. Such variation is critical considering that a previous study reported only a 9.49% difference between patients with coronary artery disease and healthy individuals [6]. To mitigate this, applying correction factors is essential. For instance, correcting fat attenuation index values using factors such as 1.24 and 1.11 for 80 kV and 100 kV, respectively, relative to the standard 120 kV can harmonize data obtained at different voltages [17].

Patient size can also affect X-ray attenuation and, consequently, fat attenuation index measurements. Larger patients exhibit slightly increased CT number, potentially leading to higher fat attenuation index values. Automatic exposure control (AEC) systems in CT scanners adjust radiation dose based on patient size to maintain image quality [18]. However, even with automatic exposure control, minimal residual variations can persist. Standardizing patient positioning and employing size-specific calibration protocols can reduce these discrepancies, ensuring more accurate fat attenuation index assessments.

Our three-material approach provides a viable strategy for investigating pericoronary adipose tissue while negating the confounding variables of tube voltage and patient size. The low RMSEs and RMSDs in our validation phase suggest that our calibration can be used for distinguishing between diseased and healthy states, even in the presence of high image noise.

A previous study has reported that fat attenuation index values differ across coronary arteries [16]. The study reported mean CT numbers of -92.4 HU for the left anterior descending artery (LAD), -88.4 HU for the left circumflex artery (LCX), and − 90.2 HU for the right coronary artery (RCA), with significant differences between these arteries [16]. These variations necessitate artery-specific reference values when interpreting fat attenuation index to accurately reflect localized inflammatory states. To account for location-dependent error, we ensured that the positions of the three-material inserts were randomly generated inside the heart, without overlap. This means that variance due to location was inherently incorporated into the calibration process.

To enhance the reliability of fat attenuation index measurements, standardizing imaging protocols is crucial. This includes using consistent tube voltages, applying appropriate correction factors, and calibrating for patient size. Implementing these strategies will improve the precision of fat attenuation index as a biomarker for vascular inflammation, facilitating its integration into clinical practice.

However, our study is not without limitations. We employed only a filtered back projection reconstruction (FBP) in our simulation; hence the impact of different reconstruction algorithms was not considered. Although the scanning parameters were calibrated to match a Canon Aquilion One CT scanner, all data were collected in a simulated ideal environment. Future studies involving physical phantoms involving the creation of water-lipid phantoms are necessary to validate the eventual clinical applicability of our results [19]. And there was no bow tie filter, a component with direct impact on image quality to reduce beam hardening effect, in the simulation. The absence of a bowtie filter in the simulation introduces systematic spatial biases in CT number measurements due to unmitigated beam hardening. In real-world CT scanners, bowtie filters are tailored to patient size (e.g., thicker filters for larger patients) to shape the X-ray beam and reduce artifacts. The simulation does not to replicate clinical conditions, leading to underestimation of central CT number. This reduces measured CT number in coronary arteries, potentially underestimating inflammation severity or water composition compared to clinical CT scans. Models using simulation data (without bowtie corrections) may fail on real CT scans, as key features (e.g., beam hardening patterns) differ.

Additionally, the effects of motion artifacts were not accounted for in the CT simulation. The exclusion of motion artifacts and reliance on a static, idealized vessel model introduce simplifications that does not represent clinical coronary CT angiography (CCTA). Real-world CCTA is affected by cardiac pulsation and respiratory motion, causing vessel blurring. The simulation’s static vessel assumption eliminates these effects, overestimating image sharpness and underestimating diagnostic uncertainty. Also, the current single-energy decomposition method is tailored for a two-material mixture, thus limiting its clinical application to two materials. The primary components of adipose tissue are water, lipid, and protein. Single-energy CT cannot resolve overlapping CT number profiles of water, lipid, and protein. Variations in protein content may be misattributed to lipid or water, skewing quantitative estimates. Dual-energy CT (DECT) leverages energy-dependent attenuation differences to distinguish materials. Water, lipid, and protein have distinct attenuation profiles at different X-ray energies (e.g., 80 kV vs. 135 kV) [10]. The previously reported coronary plaque study demonstrated that DECT can resolve water, lipid, protein, and calcium with low error (RMSE ≤ 1.5% for non-calcified plaques). This supports the principle that PVAT decomposition is technically feasible, as adipose tissue shares similar components (water, lipid, protein).

While the RMSE (0.01–0.64%) confirms the method’s accuracy in detecting a 5% water-fraction change, the RMSD (2.94–6.05%) highlights residual variability that could obscure small but clinically meaningful differences. For example, in the overlap zone between healthy (20–30%) and diseased (20–35%) ranges, an RMSD of 6% risks misclassifying borderline cases. Future work should prioritize reduction in RMSD through dual-energy CT or advanced noise reduction.

Future studies will focus on validating these simulation results using physical phantoms including cardiac motion, iterative reconstruction and dual-energy material decomposition.

## Conclusion

In conclusion, this study introduces a method for quantifying the chemical composition of perivascular adipose tissue using CT angiography, taking into account the impact of variations in tube voltage and patient sizes. Our analysis shows that tube voltage substantially changes the CT numbers of tissue components, while the influence of patient size is comparatively minor. By developing conversion factors to correct for these variations, we have provided a robust means for more accurate and consistent quantification of water volumetric fraction in perivascular adipose tissue. This ability to generate corrected CT numbers offers potential for accurate quantification of vascular inflammation, a critical precursor in atherosclerosis.

## Data Availability

No datasets were generated or analysed during the current study.
